# ERK Signals: Scaffolding Scaffolds?

**DOI:** 10.3389/fcell.2016.00049

**Published:** 2016-05-31

**Authors:** Berta Casar, Piero Crespo

**Affiliations:** Instituto de Biomedicina y Biotecnología de Cantabria, Consejo Superior de Investigaciones Científicas (CSIC) – Universidad de CantabriaSantander, Spain

**Keywords:** MAP kinase, ERK, scaffold protein, protein-protein interactions, signaling pathways

## Abstract

ERK1/2 MAP Kinases become activated in response to multiple intra- and extra-cellular stimuli through a signaling module composed of sequential tiers of cytoplasmic kinases. Scaffold proteins regulate ERK signals by connecting the different components of the module into a multi-enzymatic complex by which signal amplitude and duration are fine-tuned, and also provide signal fidelity by isolating this complex from external interferences. In addition, scaffold proteins play a central role as spatial regulators of ERKs signals. In this respect, depending on the subcellular localization from which the activating signals emanate, defined scaffolds specify which substrates are amenable to be phosphorylated. Recent evidence has unveiled direct interactions among different scaffold protein species. These scaffold-scaffold macro-complexes could constitute an additional level of regulation for ERK signals and may serve as nodes for the integration of incoming signals and the subsequent diversification of the outgoing signals with respect to substrate engagement.

Exhaustive research efforts undertaken during the past decades, have positioned the signaling module mediated by Extracellular signal-Regulated Kinases 1 and 2 (ERKs) Mitogen-Activated Protein Kinases (MAPKs), among the best known signal transduction processes ever studied. ERKs signaling cascade encompasses sequential tiers, composed of sundry types of molecular intermediaries, which become activated in response to a broad panel of intra- and extra-cellular stimuli. The ERKs cascade is generally activated at its origin by GTPases of the RAS family that subsequently switch-on, by not fully understood mechanisms, an upstream echelon constituted by MAPKKKs of the RAF family. These, at their turn, convey signals downstream by phosphorylating/activating dual-specificity MAPKKs MEK 1 and 2, ultimately responsible for the phosphorylation and the unleashing of ERKs activity (Roskoski, 2012). It is also well-known that this signaling pathway is involved in the regulation of prime physiological processes, such as cellular proliferation, differentiation, cell cycle control, development and survival, in addition to hundreds of cell- and tissue-specific events. Consequently, unregulated or aberrant ERK signaling results in multiple pathological conditions ranging from psoriasis to cancer (Robinson and Cobb, 1997; Raman et al., 2007; Shaul and Seger, 2007).

## Scaffold proteins: Orchestrators of ERK signals

ERKs pathway signal output is not solely the result of the diverse phospho-transfer reactions that occur among the constituents of the echelons that build up the route. In addition to the main players, the kinases, past research has unveiled the existence of several types of regulatory and ancillary proteins that participate at different stages of the cascade, and provide further levels of control to the signal flux. Scaffold proteins represent the most abundant, diverse and widespread category (Dhanasekaran et al., 2007). Among the regulatory proteins that associate to the constituents of a signaling cascade, the accepted requisite for considering a protein a “scaffold,” is its capacity to simultaneously bind to at least two members of such cascade, forming a functionally stable complex. The primeval evidence for a protein serving a scaffolding role in a MAPK cascade came from studies in the budding yeast *S. pombe*, in which the protein *Ste5* was found to stabilize the complex formed by *Fus3* (a MAPK), *Ste7* (MAPKK), and *Ste11* (MAPKKK) and to increase their local concentration at the tips of mating projections, in response to mating pheromones (Choi et al., 1994). The identification of mammalian scaffold proteins involved in ERKs signaling followed soon after, with the characterization of KSR1 (Kinase Suppressor of Ras) as a protein binding to CRAF, MEK1/2, and ERK1/2, forming a high-molecular weight macrocomplex (Therrien et al., 1996) whereby signaling flux through the RAS-ERK cascade was regulated. The identification of MP1 (MEK Partner 1) was next. Such scaffold was found to bind MEK1 and ERK1 but nor CRAF, and it exhibited isoform specificity as it potentiated the activation of ERK1 but not ERK2 (Schaeffer et al., 1998). Since then, the list of mammalian proteins that qualify as scaffolds for the RAS-ERK pathway has steadily expanded up to 15-odd members (Table 1). Intriguingly, none of these proteins share significant sequence homology, neither among themselves nor with Ste5 of which no mammalian homolog has been identified yet (Kolch, 2005; Dhanasekaran et al., 2007).

**Table 1:** **Locations and functions of ERK MAPK scaffolds in mammalian cells**.

**Scaffold**	**Subcellullar Localization**	**Functions**
KSR1, 2	Cytoplasm, Plasma membrane	In resting cells, KSR, Kinase Suppressor of Ras, is bound to MEK in the cytoplasm. Upon Ras activation, KSR translocates with MEK1/2 to the plasma membrane and coordinates the assembly of a multiprotein complex containing Raf, MEK, and ERK which facilitates signal transmission (Roy and Therrien, 2002; Raman et al., 2007; Lavoie and Therrien, 2015). KSR1 acts to both potentiate and attenuate ERK cascade activation (McKay et al., 2009). Deficiency of KSR1 prevents oncogenic Ras signaling in mice (Lozano et al., 2003). KSR1 acts preferentially on ERK1/2 signals emanating from PM cholesterol-rich domains (Matheny et al., 2004). cPLA_2_ activation is regulated by KSR1 when ERK1/2 are activated from lipid rafts (Casar et al., 2009a).
IQGAP 1	Cytoplasm, Focal adhesion, Cell-Cell junctions, Cytoskeleton	IQGAP1 binds B-Raf, MEK, and ERK and facilitates ERK activation by EGF (Roy et al., 2005). IQGAP1 regulates the phosphorylation of EGFr by ERK (Casar et al., 2009a). Other proteins that bind IQGAP1 include Cdc42 and Rac1, E-cadherin, β-catenin, calmodulin (White et al., 2009). IQGAP1 is over-expressed in some cancers, in some of these, high IQGAP1 levels is a sign of poor prognosis (Brown and Sacks, 2006; Jadeski et al., 2008). Blocking the interaction between IQGAP1 and ERK inhibits skin carcinogenesis driven by Ras-ERK pathway oncogenes (Jameson et al., 2013).
IQGAP 2	Cytoplasm, Cytoskeleton	IQGAP 2 associates with Cdc42, Rac1, F-Actin and calmodulin and regulates cell-cell adhesion. Deficiency of IQGAP2 predisposes to development of hepatocellular carcinoma and diabetes (Vaitheesvaran et al., 2014). Silencing of IQGAP2 contributes to gastric cancer metastasis (Jin et al., 2008).
IQGAP 3	Cytoplasm	IQGAP 3 interacts with ERK1 and enhances its phosphorylation following treatment with EGF (Nojima et al., 2008; Kunimoto et al., 2009). Overexpression of IQGAP3 promoted tumor cell growth, migration and invasion, whereas suppression of IQGAP3 in lung cancer reduces tumorigenicity (Yang et al., 2014). IQGAP 3 plays a role in FGFR1-Ras-ERK signaling, and loss of function of IQGAP3 affects both cell proliferation and cell motility (Fang et al., 2015).
Paxillin	Focal adhesion	Paxillin regulates ERK signaling at focal adhesions through other kinases such as Focal Adhesion Kinase (Ishibe et al., 2004). Paxillin—MEK-ERK complex serves as a regulator of HGF-stimulated FAK and Rac activation in the focal adhesions, thereby regulating tumor cell invasion, plasticity, and metastasis (Deakin et al., 2012). Paxillin is over-expressed in lung adenocarcinoma high-risk patients. Mutations in Paxillin have been associated with enhanced tumor growth and invasion in lung cancer (Mackinnon et al., 2011).
β arrestin 1 and 2	Cytoplasm	β-arrestins mediates ERK activation in clathrin-coated pits (DeFea et al., 2000). β-arrestins act as a scaffolds that bind C-Raf, MEK, and ERK and direct signaling to the cytosol preventing ERK translocation to the nucleus (DeWire et al., 2007; Shenoy and Lefkowitz, 2011) Dysregulation of β-arrestins expression, localization, or phosphorylation is associated with more aggressive cancer phenotypes and poorer prognosis in breast, prostate, lung, brain, and hematological tumors (Sobolesky and Moussa, 2013).
Sef 1	Golgi apparatus	Sef resides at the Golgi apparatus and binds active MEK/ERK complexes preventing ERK translocation to the nucleus but retaining it in the cytoplasm (Torii et al., 2004). Sef acts as a spatial regulator for MAPK signaling allowing phosphorylation to cytosolic substrates but not nuclear targets (Philips, 2004).
ß-Dystroglycan	Plasma membrane, Nucleus	ß-Dystroglycan interacts with MEK and active ERK, modulating ERK activity in response to integrin engagement on laminin (Spence et al., 2004). ß-Dystroglycan is involved in adhesion and adhesion-mediated signaling. Loss of the dystroglycan functions give rise to distinct disease phenotypes including muscular dystrophies and cancer (Mathew et al., 2013; Mitchell et al., 2013).
MP 1	Late Endosomes	MP1, MEK Partner-1, specifically binds to MEK1 and ERK1, but not MEK2 or EKR2 (Schaeffer et al., 1998). Over-expression of MP-1 increased ERK phosphorylation whereas down-regulation of MP-1 reduced MAPK signaling (Teis et al., 2002). MP-1 interacts with the adaptor protein p14 and enhances ERK signaling by targeting this complex to late endosomes (Teis et al., 2006)}.The MP1-p14 scaffold also enhances MEK activation by binding PAK1 to regulate cell adhesion and spreading on fibronectin (Pullikuth et al., 2005).
RKIP	Cytoplasm	In unstimulated cells RKIP, Raf Kinase Inhibitor Protein, is bound to Raf and prevents MEK phosphorylation (Park et al., 2005). Following mitogenic stimulation, RKIP dissociates from Raf to allow MEK and ERK activation (Kolch, 2005; Shin et al., 2009) RKIP functions as a metastasis suppressor in multiple solid tumor types such as prostate and breast cancer (Keller, 2004). RKIP is down-regulated in some types of cancers and is associated with resistance of cancer cells to anti-neoplastic treatments (Granovsky and Rosner, 2008).
MORG 1	Cytoplasm	MORG 1, MAPK organizer, binds C-Raf, MEK, ERK, and MP1 and facilitates ERK activation when cells are stimulated with lysophosphatidic acid or serum, but not in response to EGF (Vomastek et al., 2004).
OSBP	Cytoplasm	Oxysterol-binding protein, OSBP is a sterol-binding protein that induces ERK activation regulating vesicle transport, lipid metabolism, and signal transduction (Chen and Wang, 2004).
RGS12	Cytoplasm, Plasma membrane	Regulator of G-protein signaling, RGS associates with NGF receptor tyrosine kinase TrkA, activates Ras, B-Raf, and MEK2 and facilitates their coordinated signaling to prolonge ERK activation (Willard et al., 2007). RGS12 modulates PDGF beta receptor signaling in smooth muscle cells (Sambi et al., 2006) and regulates osteoclastogenesis in bone remodeling and pathological bone loss (Yuan et al., 2015).
archvillin	Cytoplasm	Archvillin form a complex with B-Raf, MEK, ERK and 14-3-3 in smooth muscle cells to regulate differentiation and contractility (Gangopadhyay et al., 2004, 2009).
grb10	Cytoplasm, Plasma membrane	grb10 functions as a negative regulator in the insulin –stimulted ERK signaling interacting with Raf-1 and MEK in response to IGF-I or insulin (Charalambous et al., 2003; Langlais et al., 2004; Deng et al., 2008). *Grb10* loss promotes Ras pathway hyperactivation, which promotes hyperproliferation, (Mroue et al., 2015).
dyrk1a	Cytoplasm	dyrk1a prolongs the kinetics of ERK activation by interacting with Ras, B-Raf, and MEK1 to facilitate the formation of a Ras/B-Raf/MEK1 multiprotein complex. Dyrk 1a is required for promoting or maintaining neuronal differentiation and its overexpression contributes to the neurological abnormalities observed in Down syndrome patients (Kelly and Rahmani, 2005). Dyrk1a regulates cell cycle exit, oncogene-induced senescence, and cell differentiation and acts as an oncogene in myeloid leukemias and gliomas (Abbassi et al., 2015; Lee et al., 2016).
GIT1	Cytoplasm, Plasma membrane	GIT1 acts as a scaffold that exerts spatial control of ERK1/2 activation. GIT1 colocalizes with ERK1/2 at focal adhesions. GIT1 overexpression prolongs EGF stimulation of ERK1/2, and knocking down GIT1 expression inhibits EGF stimulated ERK1/2 activity (Yin et al., 2004, 2005).

Our knowledge on the MAPKs scaffold proteins has grown significantly in recent years. We currently know that their functions extend beyond their central role as hubs for the assembly of the kinases signaling module, whereby MAPKs signals amplitude and duration are fine-tuned (Witzel et al., 2012). In this respect, several notions have gained a solid foothold in the literature. Whilst in some cases not fully demonstrated, an in others subject to vivid controversy, the concepts that follow constitute the bedrock on which our understanding of scaffold proteins has been built upon (for extensive reviews see Kolch, 2005; Dhanasekaran et al., 2007; Good et al., 2011; Witzel et al., 2012; Smith and Scott, 2013; Garbett and Bretscher, 2014), though, as usual in science, they may be far from covering all that there is to be learned about these proteins.

**Figure 1:**
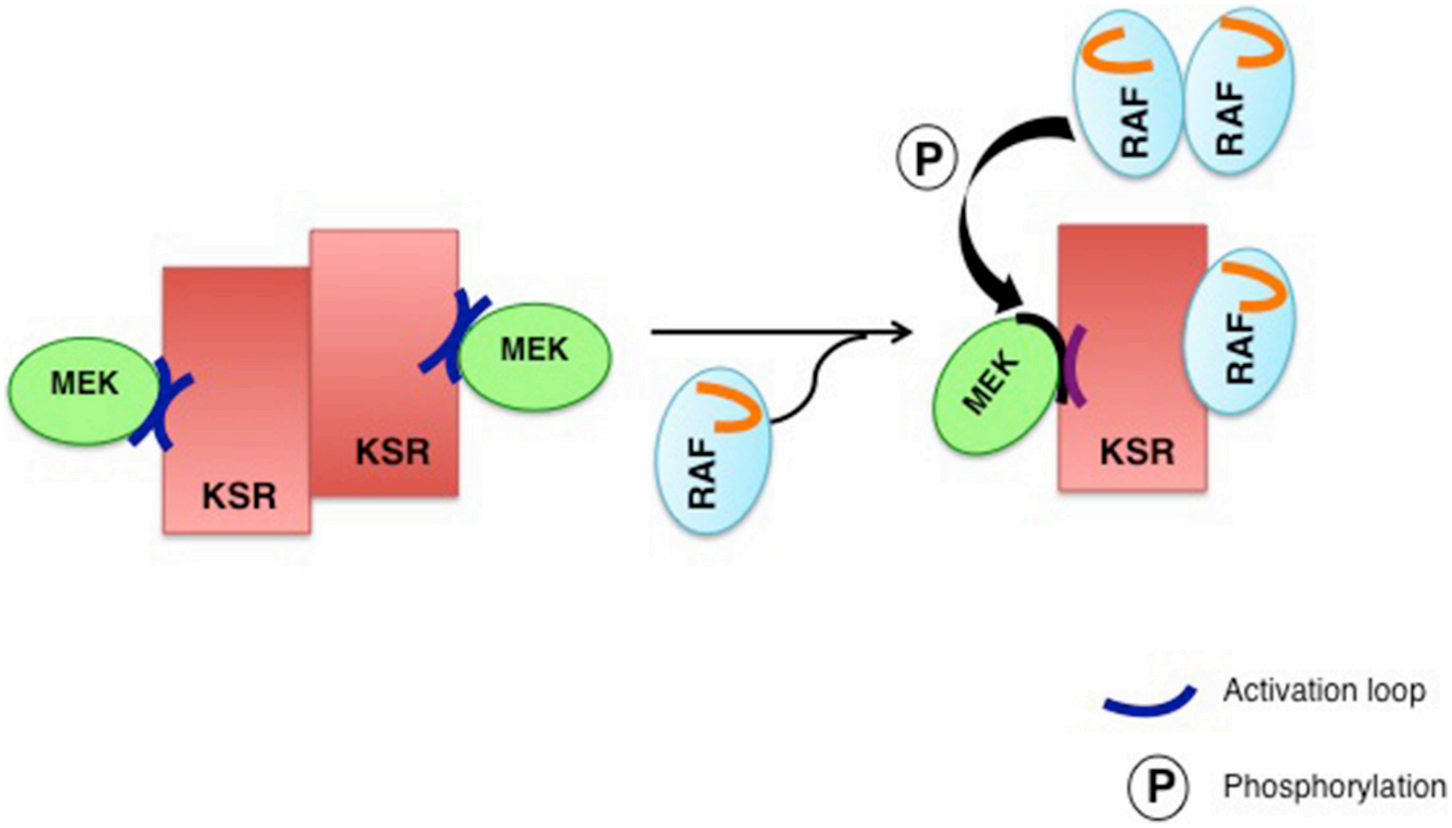
**Allosteric regulation of KSR2**. A regulatory RAF interacts with KSR in *cis* to induce a conformational switch on MEK to expose its activation loop, subject to phosphorylation by RAF in *trans*. In the KSR2–MEK1 hetero-tetramer (left), the inaccessible activation segment of MEK1 is released through the interaction of KSR2 with RAF, induced by a conformational change, allowing a “catalytic” RAF to phosphorylate MEK (right).

From the structural aspect, the prevailing model is that scaffold proteins would optimize signaling: on one hand, by tethering, thereby increasing the effective concentrations of enzymes and substrates. And on the other hand, by orienting these proteins relative to each other in order to facilitate the phospho-transfer reactions, in such a way that MAPKs will be optimally phosphorylated by the overlaying MAPKKs in a processive fashion (Scott et al., 1995; Levchenko et al., 2000). In addition, scaffolds can also enhance signal flux by acting as allosteric stimulators. For example, it has been shown that overexpression of KSR potentiates RAF activation. This is achieved via the kinase-homology domain of KSR directly binding to RAF and allosterically inducing its kinase activity (Rajakulendran et al., 2009). Furthermore, RAF interaction with KSR in *cis*, triggers a conformational switch on MEK in such a way that its activation loop is exposed and amenable for phosphorylation by RAF in trans (Brennan et al., 2011; Figure 1). These allosteric mechanisms represent additional modes of optimizing signal flux, beyond the simple tethering of the different constituents of the cascade together.

**Figure 2:**
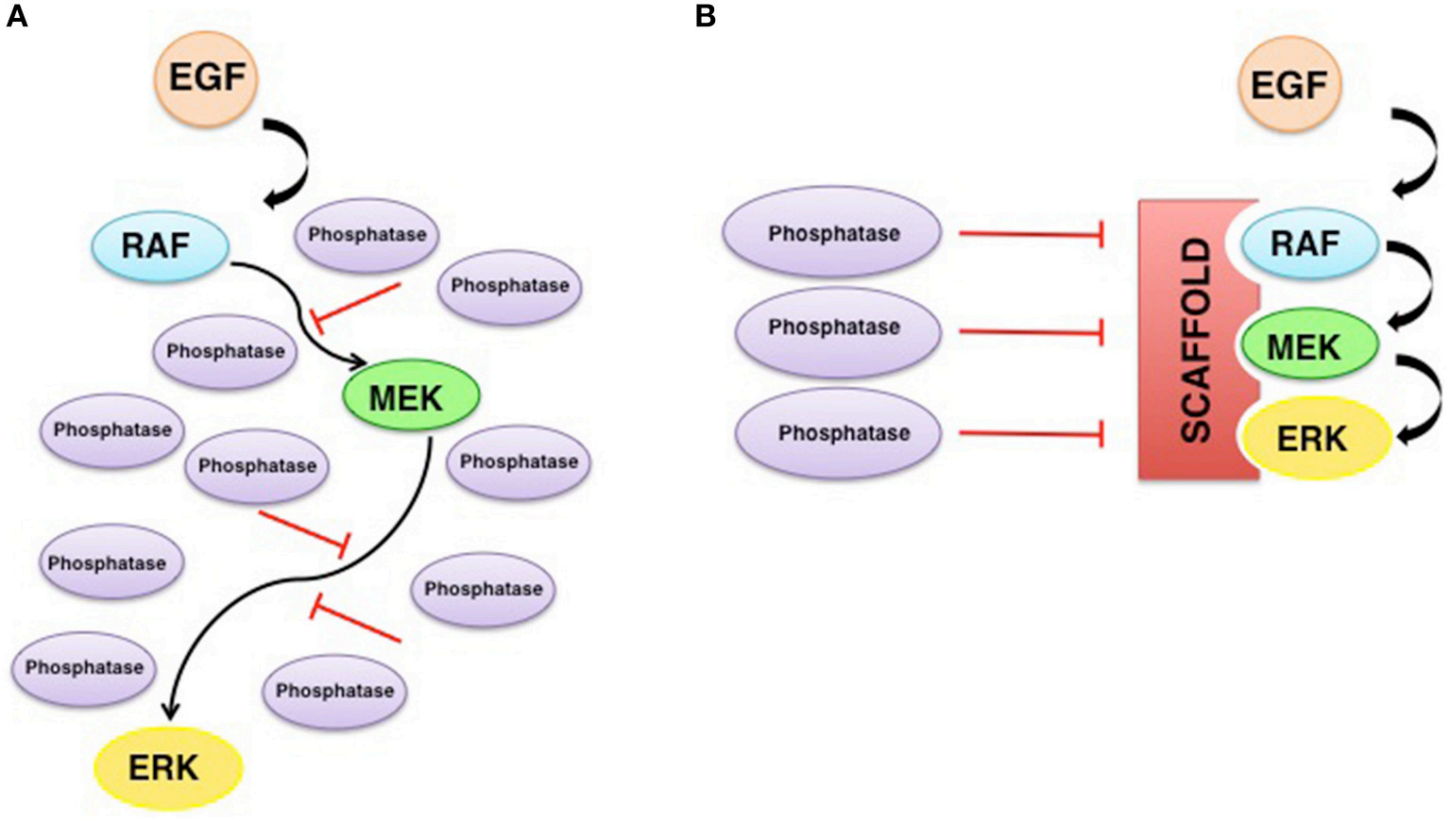
**Scaffolding promotes signal amplification in the presence of phosphatases**. High phosphatase activity in the absence **(A)** or presence **(B)** of scaffold proteins. When there are no scaffolds, the signal will be strongly down-regulated by phosphatases. Scaffold proteins enhance the local concentration of kinases and shields them from dephosphorylation, facilitating signaling.

Another deep-rooted concept is that scaffold proteins somewhat shield MAPKs from dephosphorylation, by isolating them from soluble phosphatases (Levchenko et al., 2000). A notion that impinges on a hotly debated aspect of scaffold proteins: whether they promote or impede signal amplification. Conceptually, free kinases can activate multiple targets, so the signal is amplified exponentially along the pathway. Contrarily, when tethered onto a scaffold a kinase can only phosphorylate its accompanying substrate kinase, something that would prevent signal amplification. However, if the phosphatases levels are high, a situation in which a system based on freely diffusing kinases will be strongly down-regulated, the enhanced “local” concentration effect achieved by scaffolding will result in signal amplification, by increasing the chances for a successful encounter between kinases in the midst of surrounding high levels of deactivating phosphatases (Locasale et al., 2007; Figure 2).

**Figure 3:**
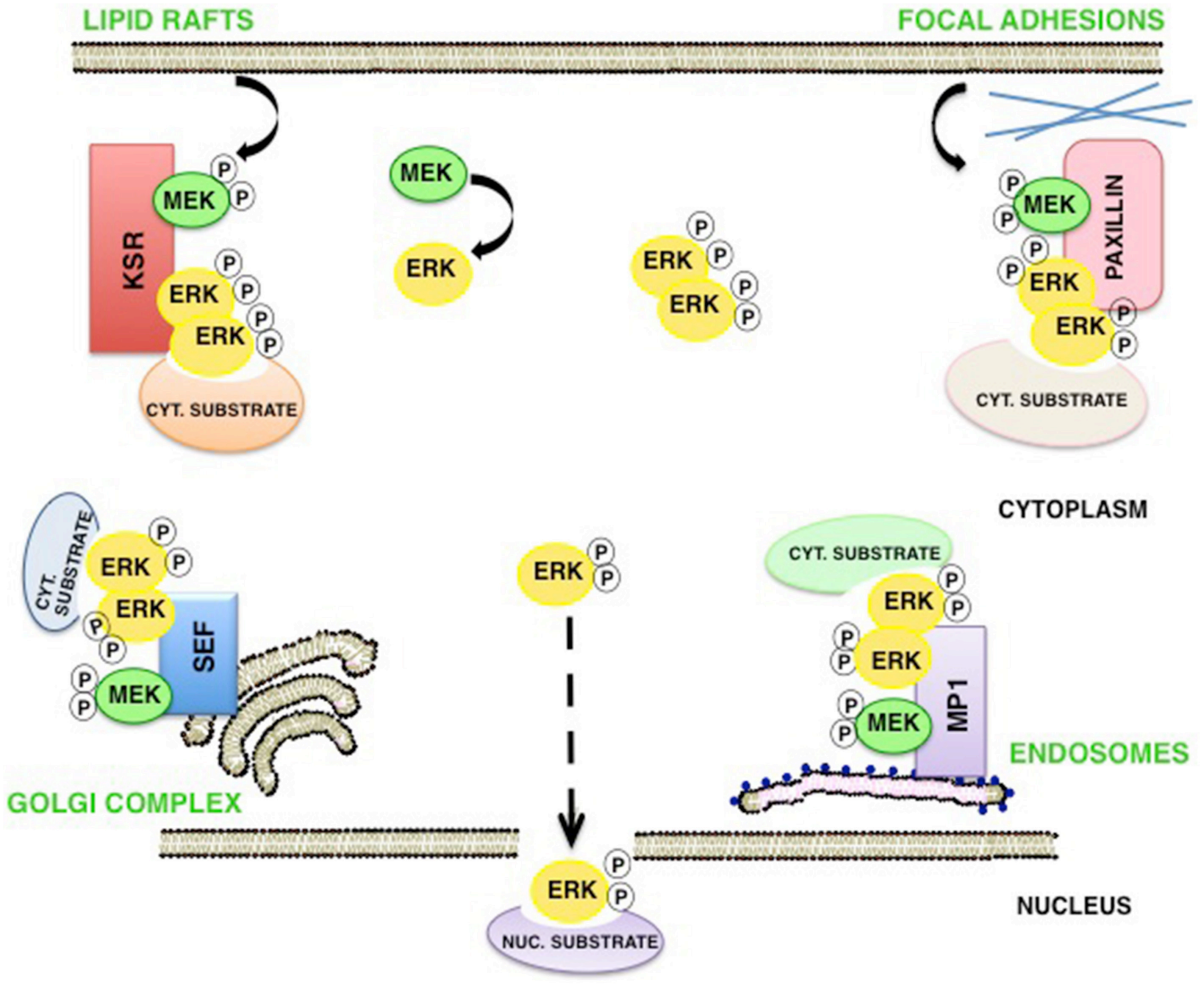
**Scaffold proteins as spatial regulators of ERK signaling**. In response to stimulation, phosphorylated ERK monomers detach from MEK and may follow three destinies: (1) translocate as monomers to the nucleus; (2) dimerize freely in the cytoplasm, and (3) specific scaffolds act as dimerization platforms in a sublocalization–specific fashion, where ERK dimers are assembled and the new complexes can interact with different cytoplasmic pools of substrates.

In addition to these ideas, it is now well established that scaffold proteins serve a central role as spatial regulators of ERKs signals, acting in a sublocalization-specific fashion. In this respect, KSR1 acts preferentially upon ERKs signals originated in lipid rafts domains (Matheny et al., 2004). MP-1 acts at endosomes (Teis et al., 2002), Sef is ERKs main scaffold at the Golgi complex (Torii et al., 2004) and Paxillin at focal adhesions (Ishibe et al., 2003). Apparently, such spatial selectivity is important for the definition of ERKs substrate specificity. It has been demonstrated that the type of membrane from which Ras signals emanate dictates which substrates are amenable to be phosphorylated by ERKs, and this is achieved through the participation of defined scaffolds depending on the origin of Ras signals (Casar et al., 2009a). The molecular mechanism whereby scaffold proteins confer substrate specificity to ERKs, is based on the fact that scaffold proteins would facilitate the formation of ERK dimers, in such a way that one ERK molecule would bind to the scaffold and the other to the pertinent substrate (Casar et al., 2008). Thus, scaffold proteins serve as ERK dimerization platforms and in so doing agglutinate the assembly of the enzymatic complexes competent for the activation of ERKs cytoplasmic substrates. In support of this notion we detected that ERKs cytoplasmic substrates such as cPLA_2_, RSK1, and PDE4, bind exclusively to ERK dimers, while a dimerization-deficient ERK2 mutant was incapable of interacting with cytoplasmic substrates (Casar et al., 2008, 2009b; Herrero et al., 2015). Accordingly, the overexpression of some scaffolds like KSR1, β-arrestin and Sef has been shown to promote ERKs functions at the cytoplasm (Sugimoto et al., 1998; DeFea et al., 2000; Tohgo et al., 2002; Torii et al., 2004) while attenuating those occurring at the nucleus. At this compartment, ERKs functions would be primarily undertaken in monomeric form (Casar et al., 2008; Figure 3).

Finally, a pivotal concept in the scaffolds theory is that for any given scaffold there exists an optimal concentration that yields maximum signal efficiency, resulting in a bell-shaped MAPK activation kinetics. In this process, sub-optimal MAPK activation occurs both when there are not sufficient scaffolds to unite all of the available MAPKs, MAPKKs, and MAPKKKs, and also when an excessive scaffold concentration scatters MAPKs, MAPKKs, and MAPKKKs in incomplete, thereby unproductive, complexes. This phenomenon has been termed “combinatorial inhibition” and “prozone effect” (Levchenko et al., 2000; Heinrich et al., 2002). Thus, it would be conceivable that by controlling fluctuations on scaffold concentrations, a biological system would find an efficient mode for regulating MAPKs signal output. Theoretically, maximum intensity for a MAPK signal would be attained only when scaffolds concentrations are at optimal levels. Thus, tilting scaffolds expression, either up or down, could be a valid means to attenuate MAPK signals. Noticeably, the expression of most scaffold proteins is rather stable and not subject to major, immediate changes in response to external stimuli and other factors that govern MAPKs activation. Something that does not preclude that alterations on scaffolds levels, when they do occur, can have profound, long-term effects on the biological outcomes mediated by MAPKs, even contributing to pathological processes. Indeed, it is well documented that some ERK scaffold proteins exhibit altered expression levels in certain types of tumors (www.oncomine.com; www.cBioportal.com).

## Coordinated scaffolds?

An intriguing aspect about ERKs scaffold proteins is that depletion, or overexpression, of any of them has dramatic effects on ERKs total signal intensity. This is somewhat counterintuitive, considering the sheer number of scaffold proteins and their, supposedly, localized mechanism of action. Conceptually, if we consider a cell in which ERK signals are tuned independently by 15 scaffold proteins, most of them acting in a sublocalization-specific fashion, any alteration on the expression of one of them should only affect ERKs total activity by about one fifteenth. However, this seems not to be the case. For example, in several studies in which KSR1 levels are down-regulated, either by gene knock-out or using RNA interference, ERK activation levels consistently drop by over 80% (Nguyen et al., 2002; Lozano et al., 2003). A similar situation is observed for IQGAP1 (Roy et al., 2004; Jameson et al., 2013), and for MP1 (Sharma et al., 2005; Teis et al., 2006), just to mention a few cases. Apparently, tampering with scaffold proteins expression levels has far more profound effects than would be expected from proteins that, supposedly, influence ERK signals locally and partially.

**Figure 4:**
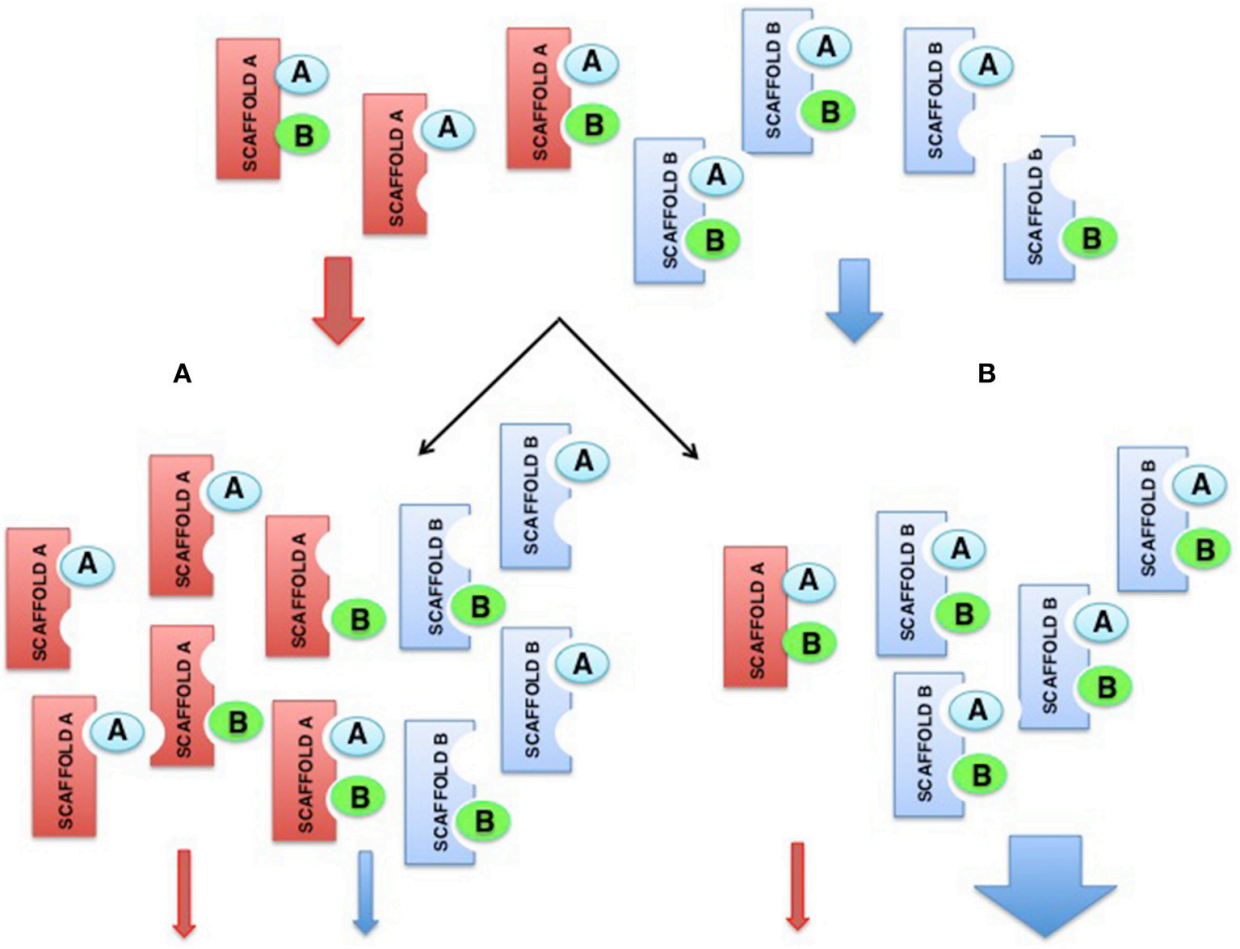
**Hypothetical model showing how alterations on scaffolds levels can impact on the functions of other scaffolds. (A)** Overexpression of the red scaffold attenuates signals from itself and from the blue scaffold, that competes for the same pools of kinases. **(B)** Depletion of the red scaffold attenuates its own signals but promotes signaling by the blue scaffold as a consequence of the increment on available kinases that increase the number of complete blue scaffold complexes.

One hypothetical explanation for this conundrum would be that scaffold proteins somehow influence the functions of other scaffold proteins. This can be easily envisioned, considering that overexpression of any scaffold should have an impact on other scaffold species that compete for the same pools of kinases, resulting in an increment on the number of incomplete scaffold complexes, for every scaffold, and therefore on less efficient signaling overall. By the same token, depletion of scaffold A could even benefit signaling as mediated by scaffold B, by reducing the competition for the same kinases and thereby increasing the number of complete scaffold B complexes (Figure 4).

Other plausible explanations have a more mechanistic basis, for example in the case of multi-domain scaffolds, such as paxillin or those of the IQGAP family, involved in multiple signal transduction events (Deakin and Turner, 2008; Smith et al., 2015). It is not unlikely that under- or overexpression of these scaffolds can have an impact on other signaling pathways that, at their turn, may induce changes in the pattern of post-translational modifications of other ERK scaffold species. Many scaffolds, for example KSR1, MP1, and SEF, are subject to phosphorylation, acetylation, ubiquitylation, and other post-translational processes (www.phosphosite.com). In most cases, the functional consequences of these modifications are completely unknown, but it is quite conceivable that, one way or another, they could have some bearing on their behavior as ERK pathway scaffolds. Indeed, it is well documented that the role played by KSR in the RAS-ERK pathway is regulated by diverse phosphorylation events (Muller et al., 2001; Razidlo et al., 2004; Dougherty et al., 2009). Specifically, KSR performance as a RAS-ERK scaffold is regulated by calcium and cAMP signals via phosphorylation (Dougherty et al., 2009; Shen et al., 2013), while these type of signals are tightly regulated by IQGAP1 (Logue et al., 2011). Thus, perturbations on IQGAP1 expression levels might impact on ERK activation both as a direct consequence of IQGAP scaffolding and indirectly, via KSR scaffolding through its regulation by Ca^2+∕^cAMP signals.

Alterations on the expression of a given scaffold could also have broader consequences than expected if scaffold proteins don't act alone mechanistically. As previously mentioned, the prevailing notion is that scaffold proteins act as autonomous entities, regulating ERK signals generated by some specific stimulus, at defined subcellular localizations. However, it cannot be discarded that different scaffolds act in a coordinated fashion to regulate flux through the ERK cascade. Indeed, evidence in mounting showing that scaffold proteins can directly associate among themselves in macromolecular complexes. Several adaptor proteins, docking proteins and scaffold proteins of diverse types are well known to interact in order to form “macro” signaling platforms (Pan et al., 2012). In the case of scaffolds for the ERK pathway, associations between different entities have been demonstrated for: IQGAP1 and MP1 (Schiefermeier et al., 2014), MP1 and MORG1 (Vomastek et al., 2004), IQGAP1 and β-arrestin2 (Feigin et al., 2014), and paxillin and GAB1 (Ren et al., 2004). And this kind of interactions seems to be important for certain cellular processes. For example, the association between IQGAP1 and MP1 appears to be critical for the regulation of focal adhesion dynamics during cellular migration (Schiefermeier et al., 2014). However, what orchestrates interactions among different scaffolds and how these interactions impact on the ability of each individual scaffold for regulating ERK signaling, are completely open questions at this moment.

**Figure 5:**
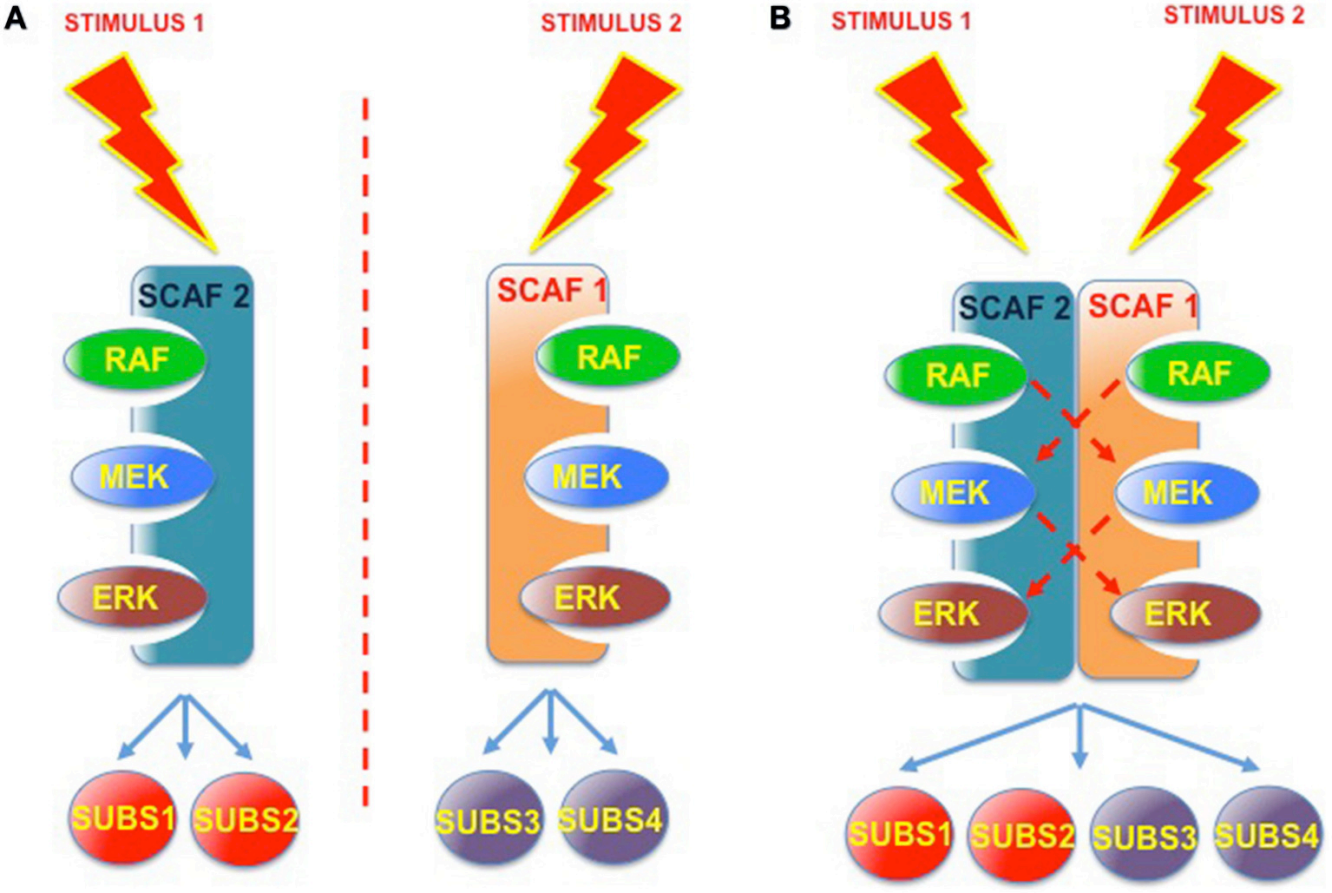
**Scaffold-Scaffold interactions as nodes for signal integration**. **(A)** Working independently, defined scaffold proteins respond to specific stimuli and convey signals to a limited number of ERK substrates. **(B)** Scaffold complexes composed of two (or more) scaffold proteins, where trans-phosphorilation among the different kinase tiers would be feasible, would facilitate signal integration, serving as nodes for various incoming signals and for the diversification of outgoing signals with respect to the number of substrates.

Overall, the unveiling of this novel kind of associations is enough to start considering the existence of higher-order “macro-scaffolding” complexes, in which the participation, maybe in some coordinated fashion, of different scaffold species could add one further degree of complexity to the regulation of ERK signals. For example, considering that signals evoked by different stimuli, or emanating from distinct sub-localizations, could engage diverse scaffolds and target distinct pools of substrates (Casar et al., 2009b). It is conceivable that complexes formed by the association of two different scaffold proteins, and competent for “trans-phosphorylation” between the different kinase tiers, may serve as nodes for the integration of incoming, distinct spatially-defined signals, and for the subsequent diversification of outgoing signals with respect to substrate specificity (Figure 5). As an example, MORG facilitates ERK activation as evoked by serum but not by EGF (Vomastek et al., 2004). Contrarily, MP1 responds to EGF (Teis et al., 2002) but not to serum (Sharma et al., 2005). If MORG and MP1 directed ERK to different sets of substrates, EGF or serum stimulation would result in activation of just a narrow collection of substrates. However, a MORG-MP1 association would make available the whole spectrum of substrates both to serum- and to EGF-induced ERK signals, provided that trans-phosphorylation occurred between both scaffold complexes.

**Figure 6:**
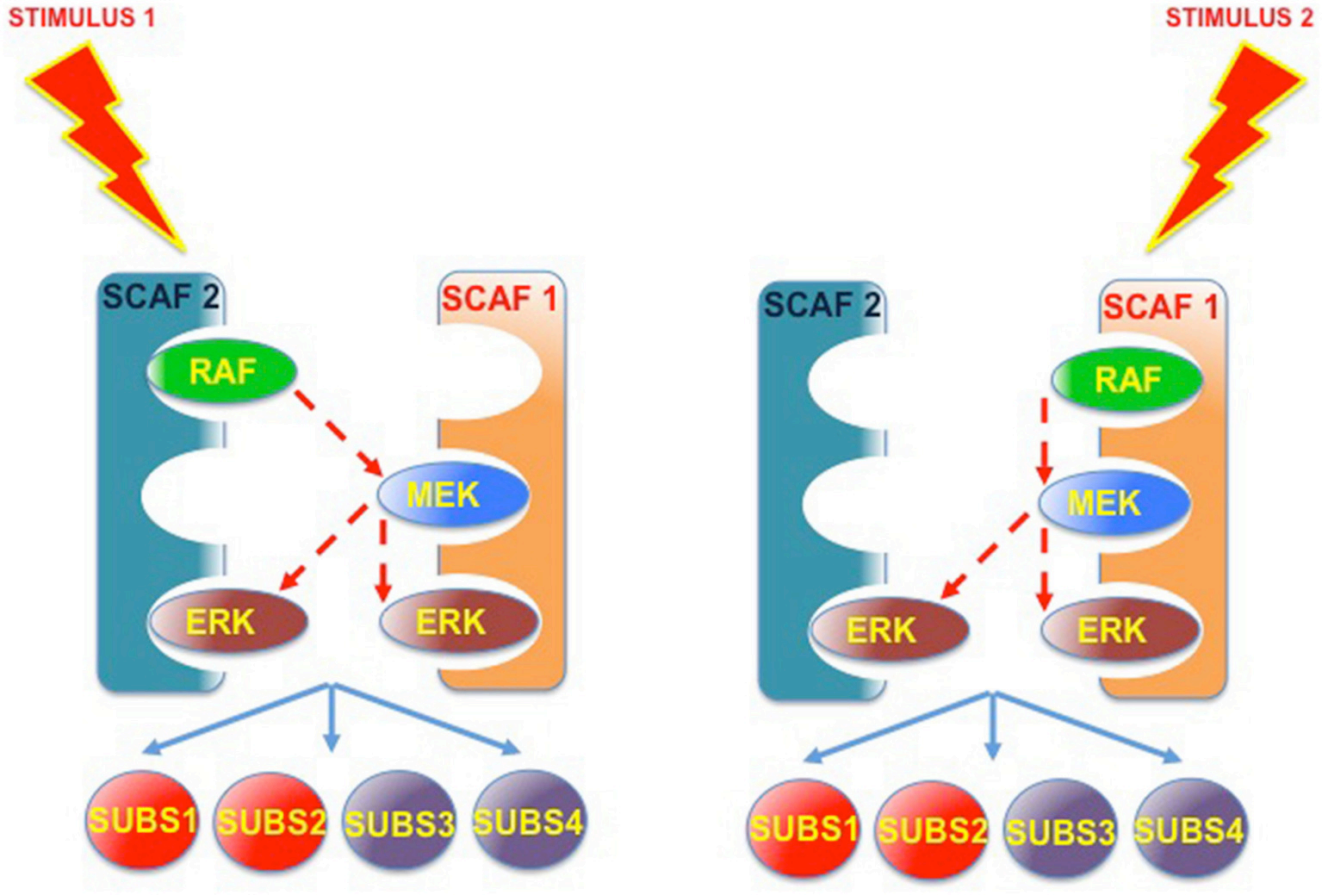
**Scaffold-Scaffold interactions may compensate deficiencies in kinases, facilitating signaling**. Provided that trans-phosphorylation was possible, complexes formed by two (or more) partially occupied scaffolds would be able to complement each other's kinase deficiencies, so incomplete scaffold complexes, apparently impaired for supporting efficient signaling, would be capable of improving the flux of signals.

Furthermore, the possibility exists that an incomplete scaffold complex (missing one or more kinases) could interact in *trans* with another type of scaffold, also partially occupied, to allow trans-phosphorylation. This would enable the different scaffolds to complement, and compensate, each other's kinase deficiencies (Figure 6). In this fashion, incomplete scaffold complexes, apparently impaired for supporting efficient signaling, would be capable of contributing to the flux of signals. Thus, if scaffolds were to function cooperatively, signal optimization could be possible under situations in which different scaffold species, acting on their own, would be at a disadvantage. For example, when the levels of some kinase are limiting. This cooperation would be particularly advantageous in those cases in which the collaborating scaffolds exhibit markedly different affinities for the limiting kinase.

Such interdependence among different scaffold species, could offer a plausible explanation for the dramatic consequences on ERK signaling, frequently observed when tampering with the expression levels of most scaffold proteins. If proven to be correct, associations among different scaffold proteins will add one further degree of regulation for an already tightly regulated cascade and could provide a novel means for manipulating ERK signals, even with therapeutic purposes.

## Author contributions

Both PC and BC have made substantial, direct, and intellectual contribution to the work, and approved it for publication.

### Conflict of interest statement

The authors declare that the research was conducted in the absence of any commercial or financial relationships that could be construed as a potential conflict of interest.
